# Bacterial-mediated synthesis and characterization of copper oxide nanoparticles with antibacterial, antioxidant, and anticancer potentials

**DOI:** 10.3389/fbioe.2023.1140010

**Published:** 2023-03-06

**Authors:** Seyedehsaba Talebian, Bahar Shahnavaz, Masoud Nejabat, Yasaman Abolhassani, Fatemeh B. Rassouli

**Affiliations:** ^1^ Department of Biology, Faculty of Science, Ferdowsi University of Mashhad, Mashhad, Iran; ^2^ Department of Medical Biotechnology and Nanotechnology, Faculty of Medicine, Mashhad University of Medical Science, Mashhad, Iran; ^3^ Novel Diagnostics and Therapeutics Research Group, Institute of Biotechnology, Ferdowsi University of Mashhad, Mashhad, Iran

**Keywords:** *stenotrophomonas* sp. BS95, copper oxide nanoparticles, antimicrobial activity, antioxidant effects, anticancer properties

## Abstract

The application of novel bacterial strains for effective biosynthesis of nanoparticles minimizes negative environmental impact and eliminates challenges of available approaches. In the present study, cell-free extract of *Stenotrophomonas* sp. BS95. was used for synthesis of copper oxide nanoparticles (CuONPs). Characterization of crude and calcined CuONPs was carried out by UV-vis spectroscopy, X-ray diffraction (XRD), fourier transform infrared (FTIR) spectroscopy, zeta potential, dynamic light scattering, field emission scanning electron microscopy, transmission electron microscopy, and atomic force microscopy. Afterward, biogenic CuONPs were evaluated for antibacterial, antioxidant, and cytotoxic effects using broth micro-dilution method, DPPH assay and alamarBlue assay, respectively. Finally, molecular mechanisms behind anticancer effects of CuONPs was ascertained by real time PCR. UV-vis absorbance spectra registered surface plasmon resonance peaks at 286 nm and 420 nm for crude and calcined CuONPs, respectively. FTIR spectra exhibited bands associated with organic functional groups of bacterial proteins, confirming capping and functionalization of CuONPs. The average crystallite size of crude and calcined CuONPs was determined as 18.24 and 21.3 nm by XRD, respectively. The average zeta potentials of crude and calcined CuONPs were as −28.57 ± 5.13 and −29.47 ± 4.78 mV, respectively, indicating their high stability. Electron microscopy revealed that crude and calcined CuONPs were roughly spherical particles with an average size of 35.24 ± 4.64 and 43.68 ± 2.31 nm, respectively. Biogenic CuONPs induced antibacterial effects with minimal inhibitory concentrations ranging from 62.5 to 1,000 μg/ml against Gram-negative and Gram-positive strains. The antioxidant activity of crude and calcined CuONPs was found to be 83% ± 2.64% and 78% ± 1.73%, respectively. More intriguingly, CuONPs exerted considerable cytotoxic effects on human colon and gastric adenocarcinoma cells, while induced low toxicity on normal cells. Anticancer effects of biogenic CuONPs were confirmed by significant changes induced in the expression of apoptosis-related genes, including *P53*, *BAX*, *BCL2* and *CCND1*. Hence, biosynthesized CuONPs could be considered as potential antimicrobial, antioxidant and anticancer agents.

## Introduction

Nanotechnology is as an evolving interdisciplinary research field that focuses on nanoparticles (NPs) with improved size-dependent features, such as robustness of the colloidal moiety, great surface area and high bioavailability to name a few (Pan et al., 2021). Conventionally, synthesis of NPs can be performed by physical, chemical and mechanical approaches, however, high cost and toxicity of these methods have shifted fabrication of NPs toward biological systems (Chandrasekaran et al., 2020; Lahiri et al., 2021). Among all microorganisms, bacteria are attractive candidates to synthesize NPs because of remarkable benefits like high stability, short generation time, mild experimental condition, easy culture, resistance to most toxic heavy metals and their ability to produce sustainable NPs at a large scale (Yusof et al., 2019; Waris et al., 2021).

Unique characteristics of metal oxide NPs made them suitable candidates for various commercial and domestic applications, such as energy harvesting, food processing and environmental protection (Tsuzuki, 2021). In addition, there is a growing interest to develop nano-scale pharmaceuticals for management of global health concerns. For instance, antibiotic resistance crisis, which has placed a substantial clinical and financial burden on healthcare systems, can be controlled by antibacterial potential of NPs. Furthermore, increasing incidence and mortality rate of cancer, which is due to poor diagnosis and low specificity of common chemotherapeutics, can be reversed by novel nano-based agents (Pearce et al., 2017; Fu et al., 2018; Zhang et al., 2018).

Copper oxide nanoparticles (CuONPs) have attracted much attention because of appropriate redox potential, high specific surface area, and excellent stability in different solutions (Nagajyothi et al., 2017; Verma and Kumar, 2019). The aim of present study was to synthesize CuONPs by a toxic-free, rapid, and eco-friendly approach and evaluate its biological activities. To do so, CuONPs were first synthesized by *Stenotrophomonas* sp. BS95, and then characterization was carried out by well-established techniques; Optical properties were defined by ultraviolet-visible (UV-vis) spectroscopy and fourier transform infrared (FTIR) defined functional groups. X-ray diffraction (XRD) was used to determin the crystallite size, while physical stability and surface charge were measured by zeta potential. Analysis of hydrodynamic particle size was carried out by dynamic light scattering (DLS) and electrone microscopy was used to study various surface phenomena such as morphology and roughness. Then after, crude and calcined CuONPs were assessed for antibacterial, antioxidant, and cytotoxic effects using broth micro-dilution method, DPPH assay and alamarBlue assay, respectively. Finally, molecular mechanisms behind anticancer effects of biogenic CuONPs was unraveled by real time polymerase chain reaction (PCR).

## Materials and methods

### Biosynthesis of CuONPs by hydrothermal cell lysate supernatant

Synthesis of CuONPs in the present study was carried out using bacterial cell lysate supernatant (CLS), as previously described (Nakhaeepour et al., 2019). A cold-tolerant bacterium, namely, *Stenotrophomonas* sp. BS95 was isolated from alpine soil samples collected in western Iran, and identified using 16s rRNA gene sequencing analysis. The data presented in this study are deposited in the GenBank repository, accession number OQ253458. To synthesize CuONPs, this strain was cultured in tryptic soy broth (TSB) medium (Merck) and incubated in a shaking incubator at 28°C and 150 rpm for 72 h. Afterward, the culture medium was centrifuged at 7,500 rpm for 15 min and the pellet was washed with 1 mM NaCl solution (Merck). Then, the cell pellet was resuspended in distilled H_2_O and after 24 h incubation, it was placed in an ultrasonic bath sonicator for 20 min to obtain the cell lysate. Upon centrifugation at 5,000 rpm for 20 min, the supernatant was added to 0.01 M copper (II) sulfate pentahydrate (CuSO_4_.5H_2_O) and heated at 121°C for 20 min. Followed by centrifugation at 10,000 rpm for 10 min, biogenic CuONPs were obtained and washed with distilled H_2_O and ethanol. Then, the purified NPs were dried in a vacuum oven at 80°C for 4 h to achieve crude CuONPs, and calcined CuONPs were obtained after incubation in a muffle furnace (470°C) for 4 h.

## Characterization of synthesized CuONPs

### UV-vis spectroscopy

To define optical properties of crude and calcined CuONPs, UV-vis spectroscopy was used (Ssekatawa et al., 2022). In this regard, 1 mg of each sample was dispersed in distilled H_2_O and dispensed into different cuvettes. Optical properties of samples were then obtained by UV-vis spectrophotometer (Shimadzu UV-1700, Japan), scanning at a resolution of 1 nm between 200 and 800 nm ranges.

### FTIR spectroscopy

The organic functional groups of crude and calcined CuONPs were identified by FTIR spectroscopy (Sarkar et al., 2020). In this regard, 2 mg of biogenic CuONPs and 2 g of potassium bromide (KBr) were mixed and compressed to obtain translucent circular pellets. Then, samples were scanned through 4,000 to 400 cm^-1^ wavenumber range and a resolution of 4 cm^-1^ for at least 32 scans per sample using Thermo Nicolet 6700 FTIR spectrometer (Nicolet Avatar, Madison, WI, United States of America). To note, KBr pellet was used as control.

### XRD analysis

To confirm crystallinity of crude and calcined CuONPs, XRD analysis was performed (Nakhaeepour et al., 2019). To do so, GNR Explorer X-ray diffractometer (Italy) fitted with Cu-Kα radiation (*λ* = 1.5418 A°) and scanning from 2θ = 20°–80°, with a voltage of 40 kV, current of 30 mA and integration time of 0.2 s/step was used. Obtained data was visualized in OriginPro 2019b software, and validated by standard CuONPs 2θ values from the International Center for Diffraction Data (ICDD) database. The average crystallite size was calculated using the following formula:

d=0.9 λ / β cos⁡θ

in which d is the average crystallite size, *ß* is full peak width at half maximum, *λ* is the wavelength of X-ray (1.5418 Å) and θ is the 2θ angle in peak.

### DLS and zeta potential

The average size and stability of crude and calcined CuONPs were determined at neutral pH and room temperature as previously described (Sarkar et al., 2020). Briefly, to evaluate the average size distribution, 100 μg/ml of each sample was dispersed in ethanol and sonicated for 5 min. Afterward, samples were examined by DLS analyzer (vasco3. Cordouan, France) for three times, and zeta potential was determined by an electrophoretic light scattering instrument (Zeta Compact, CAD, France).

### FESEM, TEM and AFM analysis

To determine the particle size distribution and nanostructure of crude and calcined CuONPs, FESEM was used (Ali et al., 2020). In summary, each sample was spread onto an aluminum tape and coated with gold to become a conductor. Micrographs were taken at different magnifications using FESEM (Mira 3-FEG TESCAN, Czech Republic), operating at around 30 kV accelerating voltage. Morphological and topographical characteristics of biogenic CuONPs were also studied by TEM (Ssekatawa et al., 2022). In this regard, 1 mg/ml of each sample was dispersed in ethanol, sonicated and finally loaded on copper grid thin films. Micrographs were obtained by TEM (912AB, LEO, Germany) operating at around 100 kV accelerating voltage. The particle size distribution (PSD) plots were obtained by determining the size of 50 particles for each sample using ImageJ software.

The surface morphology of crude and calcined CuONPs was investigated by AFM (Barani et al., 2021). To do so, NSC15-type silicon probes with the radius of tip curvature less than 10 nm were used and samples were analyzed by AFM device (Brisk model, Ara Research, Iran). The height of each sample was finally estimated in scanning areas of 1 × 1 μm.

### Antibacterial activity

The antibacterial activity of biogenic CuONPs against pathogenic bacteria including *Bacillus subtilis* PTCC 1023*, Staphylococcus aureus* ATCC 25923*, Pseudomonas putida* KT2440 and *Escherichia coli* PTCC 1860 was evaluated using broth micro-dilution method (Binesh et al., 2021) The suspension of 1.5 × 10^8^ CFU/mL bacteria (according to the 0.5 McFarland standard) was prepared in nutrient broth (NB, Merck). To assess minimum inhibitory concentration (MIC), 100 µl of CuONPs with serial dilutions (500–1.9 μg/ml) and 10 µl of pathogenic bacteria were transferred to each well of 96-well plates containing 100 μL Muller-Hinton broth medium (MHB, Merck) and incubated at 37°C in a shaker incubator for 18 h. The absorbance was then recorded at 630 nm using a spectrophotometer (Stat Fax 2100, England). To evaluate minimum bactericidal concentration (MBC), 5 µl from each dilution was spread on Muller-Hinton agar (MHA, Merck) plates and incubated at 37°C for another 24 h.

### Analysis of antioxidant activity

The antioxidant activity of crude and calcined CuONPs was assessed by measuring their capability to scavenge synthetic stable radicals of 2,2-diphenyl-1-picrylhydrazyl (DPPH), as previously reported (Barani et al., 2021). To do so, 0.14 mM DPPH in methanol was added to each well of 96-well plates containing different concentrations of CuONPs (31.2, 62.5, 125, 250, 500, and 1,000 μg/ml) and incubated at 37°C for 30 min, while ascorbic acid was used as a standard solution. Finally, the absorbance (A) was recorded at 517 nm using spectrophotometer (Awarness), and free radical scavenging activity of CuONPs was calculated using the following formula:

$$DPPH\;radical\;scavenging\;activity\;\left(\%\right)=\left(\frac{AS-\left(AT-AB\right)}{AS}\right)\times 100$$

in which AT is the absorbance of test wells, AB is the absorbance of blank wells, and AS is the absorbance of standard solution.

### Cell culture, treatment and viability assay

Human colon and gastric adenocarcinoma cells (LoVo and MKN-45 cell lines, respectively) along with human dermal fibroblasts (HDF cell line) were purchased from Pasteur Institute (Tehran, Iran). MKN-45 and HDF cells were cultured in Dulbecco’s modified Eagle’s medium (DMEM, Biowest), whereas LoVo cells were grown in Roswell Park Memorial Institute-1640 (RPMI-1640, Biowest). All media were supplemented with 10% fetal bovine serum (FBS) (Biowest) and 1% penicillin-streptomycin (Biowest). Cells were maintained at 37°C in normoxic (95% and 5% CO_2_ in air) and hypoxic (93% N_2_, 5% CO_2_ and 2% O_2_) conditions.

To evaluate cytotoxic effects of CuONPs and determine the half maximal inhibitory concentration (IC_50_) values, alamarBlue assay was performed (Movaffagh et al., 2021). To do so, LoVo and MKN-45 cells were seeded at a density of 14,000 cell/well, in each well of 96-well plates, while HDF cells were seeded at a density of 10,000 cell/well. After 24 h incubation, cells were treated with 50, 100, and 200 μg/ml crude and calcined CuONPs, while untreated cells were considered as control. At the end of treatments (24 h), alamarBlue solution (Sigma-Aldrich) was added to each well (20 µl/well) followed by 2 h incubation at 37°C. Then, the absorbance (A) of each well was recorded at 600 nm using spectrophotometer (BioTek), and the viability of cells was calculated based on the following equation:

$$Cell\;viability\;\left(\%\right)=\left[100\;–\left(\frac{AT\;–\;AU}{AB-AU}\right)\right]\times 100$$

in which AT is the absorbance of treated cells, AU is the absorbance of untreated cells, and AB is the absorbance of blank control.

### Gene expression analysis

To assess the effects of biogenic CuONPs on the expression of apoptosis-related genes, real time PCR was applied (Mirzaei et al., 2022). In summary, total cellular RNA was extracted from LoVo cells treated with 100 μg/ml crude and calcined CuONPs, as well as untreated cells, using a total RNA isolation kit (DENAzist Asia). RNA purity was then evaluated by spectrophotometer at 260 and 280 nm (Nanodrop 2000 Thermo). For synthesis of cDNAs, M-MuLV reverse transcriptase (Parstous) was used according to the manufacturer’s instruction. The validity of amplified cDNAs was then confirmed by PCR using *TBP* primers and final products were loaded on 1.5% agarose gel for electrophoresis. Real time PCR was conducted in an iQ5 real-time PCR detection system (Bio-Rad) using SYBR green master mix (BioFact) and specific primers listed in Table 1. To compare the level of gene expression, TBP transcripts were used as internal control and normalized values were plotted as relative fold change over untreated cells. PCR cycling conditions were as follows: 95°C for 5 min [95°C for 20 s, 58°C for 30 s, 72°C for 30 s] (35 cycles) for *P53*, *BAX*, *BCL2* and *CCND1* primers.

**TABLE 1:** List of primers, their sequence, and product length used in the present study.

Gene name	Forward (5′ to 3′)	Reverse (5′ to 3′)	Product size (bp)
*TBP*	ACA​ACA​GCC​TGC​CAC​CTT​A	GAA​TAG​GCT​GTG​GGG​TCA​GT	120
*P53*	GTT​CCG​AGA​GCT​GAA​TGA​GG	TTA​TGG​CGG​GAG​GTA​GAC​TG	123
*BAX*	GGA​CGA​ACT​GGA​CAG​TAA​CAT​GG	GCA​AAG​TAG​AAA​AGG​GCG​ACA​AC	150
*BCL2*	GAT​GAC​TGA​GTA​CCT​GAA​CCG	CAG​AGA​CAG​CCA​GGA​GAA​ATC	124
*CCND1*	TGA​AGG​AGA​CCA​TCC​CCC​TG	TGT​TCA​ATG​AAA​TCG​TGC​GG	151

### Statistical analysis

The data were analyzed by one-way ANOVA and Dunnett’s multiple comparison tests using GraphPad Prism version 8.4.3 software. Values were expressed as mean ± SD, and *p* values less than 0.05, 0.01, 0.001 and 0.0001 were considered to be statistically significant.

## Results

### Biosynthesis of CuONPs

**FIGURE 1:**
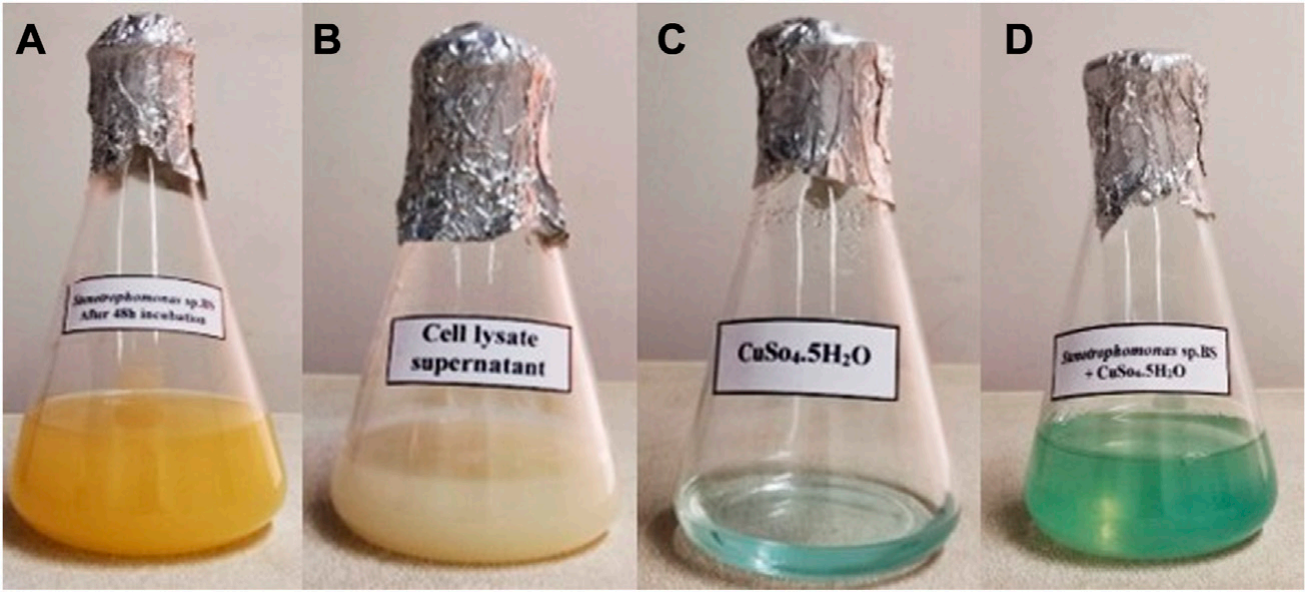
Visual detection of biosynthesized CuONPs. Four conical flasks containing *Stenotrophomonas* sp. BS95 after 48 h incubation **(A)**, bacterial CLS **(B)**, CuSO_4_ solution **(C)**, and biogenic CuONPs **(D)**.

In the present study, synthesis of CuONPs was carried out using CLS of a psychrotolerant *Stenotrophomonas* species. In this approach, complicated downstream processes were not required and thus, the risk of microbial contamination was low. The biosynthesis of CuONPs was validated by monitoring four flasks containing *Stenotrophomonas* sp. BS95 after 48 h incubation, bacterial CLS, CuSO_4_ solution and the reaction mixture of bacterial CLS with CuSO_4_ (Figure 1). The instant precipitation of green aggregates, which did not change over 24 h incubation, indicated the formation of CuONPs.

### Optical properties of biogenic CuONPs

**FIGURE 2:**
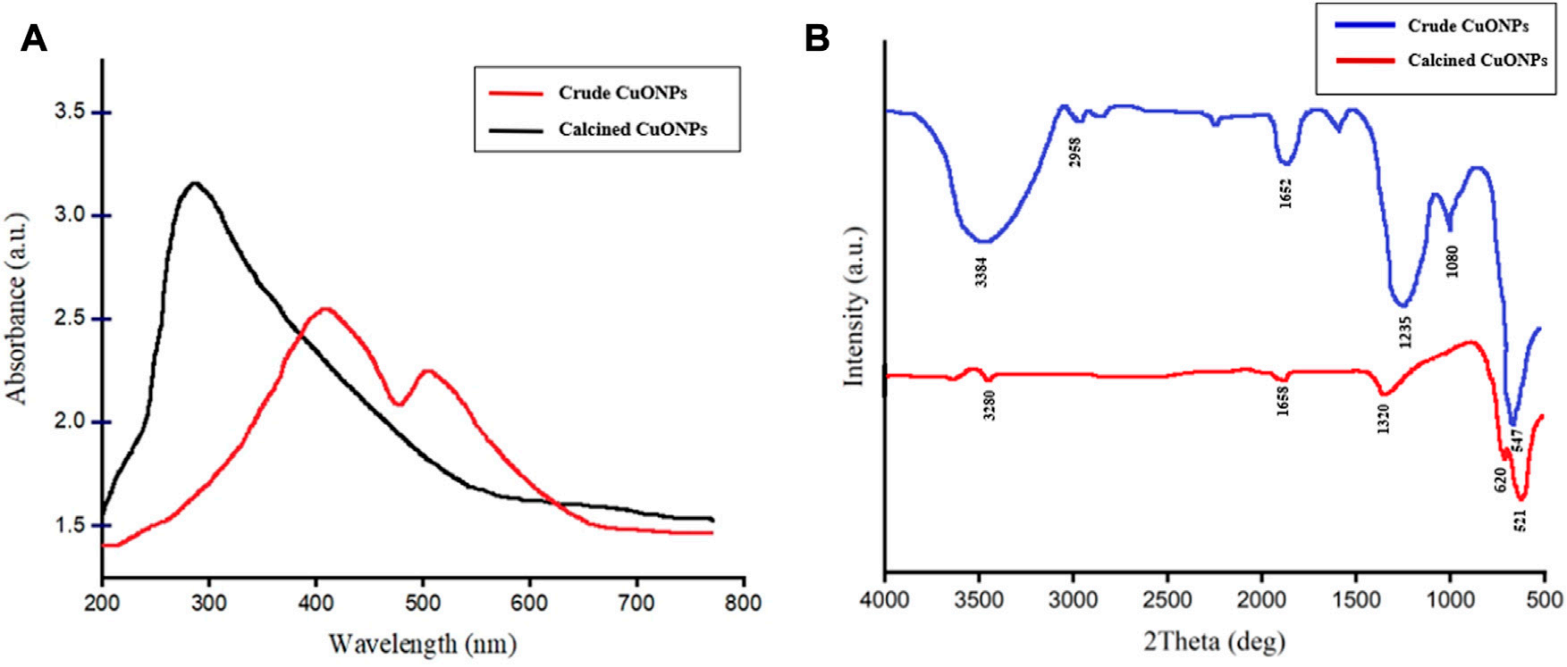
The UV-vis spectrum of crude and calcined CuONPs **(A)**; maximum absorbance bands were observed at 286 nm and 420 nm, respectively, FTIR spectrum of crude and calcined CuONPs **(B)**.

UV-vis spectroscopy was carried out to obtain optical properties of crude and calcined CuONPs. As presented in Figure 2A, surface plasmon resonance (SPR) peaks were recorded at 286 and 420 nm, which were assigned to efficient bio-reduction of CuSO_4_ to CuONPs. Since the shift of UV-vis absorbance toward short wavelengths is mainly attributed to decrease in the NP size, the left shift observed in UV spectrum was associated with the small size of our biogenic crude CuONPs.

### FTIR analysis

The FTIR spectra of crude and calcined CuONPs revealed similar absorbance bands within the wavenumber range of 3,200–3,600, 1,652 and 1,235–1,360 cm^-1^ (Figure 2B). As shown, three distinct bands were registered for crude CuONPs in the range of 2,958, 1,080 and 547 cm^−1^, while two unique absorbance bands were recorded for calcined CuONPs in the range of 521 and 620 cm^−1^.

### Analysis of XRD pattern

**FIGURE 3:**
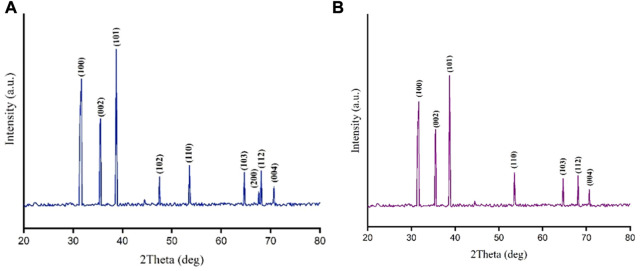
XRD patterns for crude **(A)** and calcined **(B)** CuONPs.

Completely similar to the standard pattern of CuO nanocrystals (JCPDS File No: 01-080-1917), analysis of XRD patterns revealed that all peaks representing CuONPs were present in our biogenic crude and calcined NPs (Figure 3). Bragg peaks positioned at 2θ values of 32.5°, 35.7°, 38.7°, 46.2°, 53.5°, 65.8°, 66.2°, 68.08° and 72.3° were registered for crude CuONPs, corresponded to the planes of (100), (002), (101), (102), (110), (103), (200), (112) and (004), respectively. However, diffraction peaks observed at 2θ values of 32.18°, 35.19°, 38.6°, 52.9°, 65.3°, 68.2° and 71.8° were registered for calcined CuONPs, assigned to crystal planes of (100), (002), (101), (110), (103), (112) and (004), respectively. The average crystallite size, measured using the Debye–Scherrer equation, was as 18.24 nm and 21.3 nm for crude and calcined CuONPs, respectively.

### Measurement of stability and particle size

**FIGURE 4:**
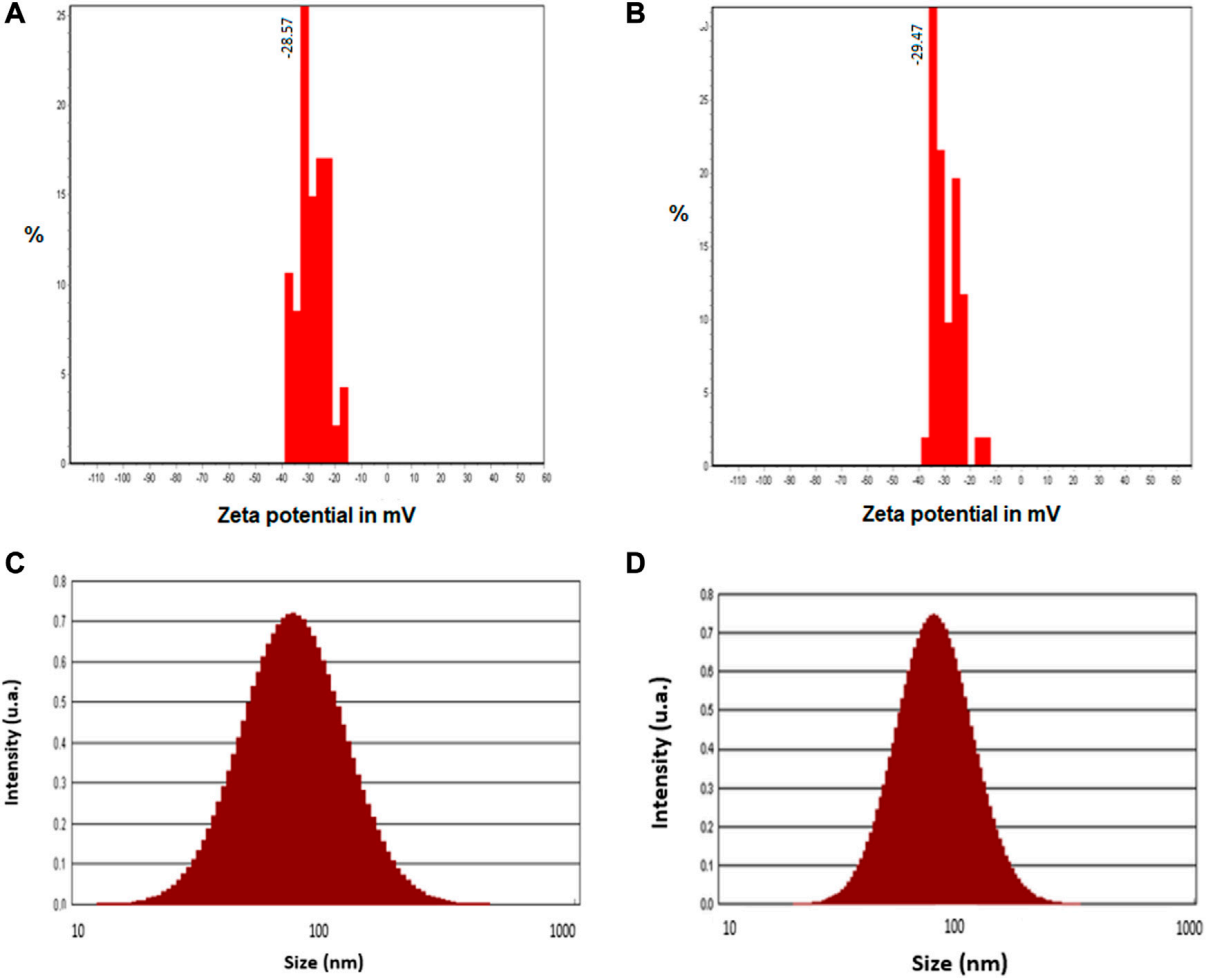
Zeta potential histograms **(A, B)** and particle size distribution pattern **(C, D)** of crude **(A, C)** and calcined **(B, D)** CuONPs.

The average zeta potentials of crude and calcined CuONPs were as −28.57 ± 5.13 mV and −29.47 ± 4.78 mV, respectively, indicating their high stability (Figures 4A, B). The size distribution histograms obtained from DLS analysis showed that the size of the crude CuONPs ranged from 25 to 110 nm with a mean distribution diameter of 55.92 nm, and from 38 to 128 nm with an average of 68.35 nm for calcined CuONPs. In addition, the calculated polydispersity index (PDI) were as 0.26 nm and 0.13 nm for crude and calcined CuONPs, respectively (Figures 4C, D).

### Nanostructure analysis by FESEM, TEM and AFM

**FIGURE 5:**
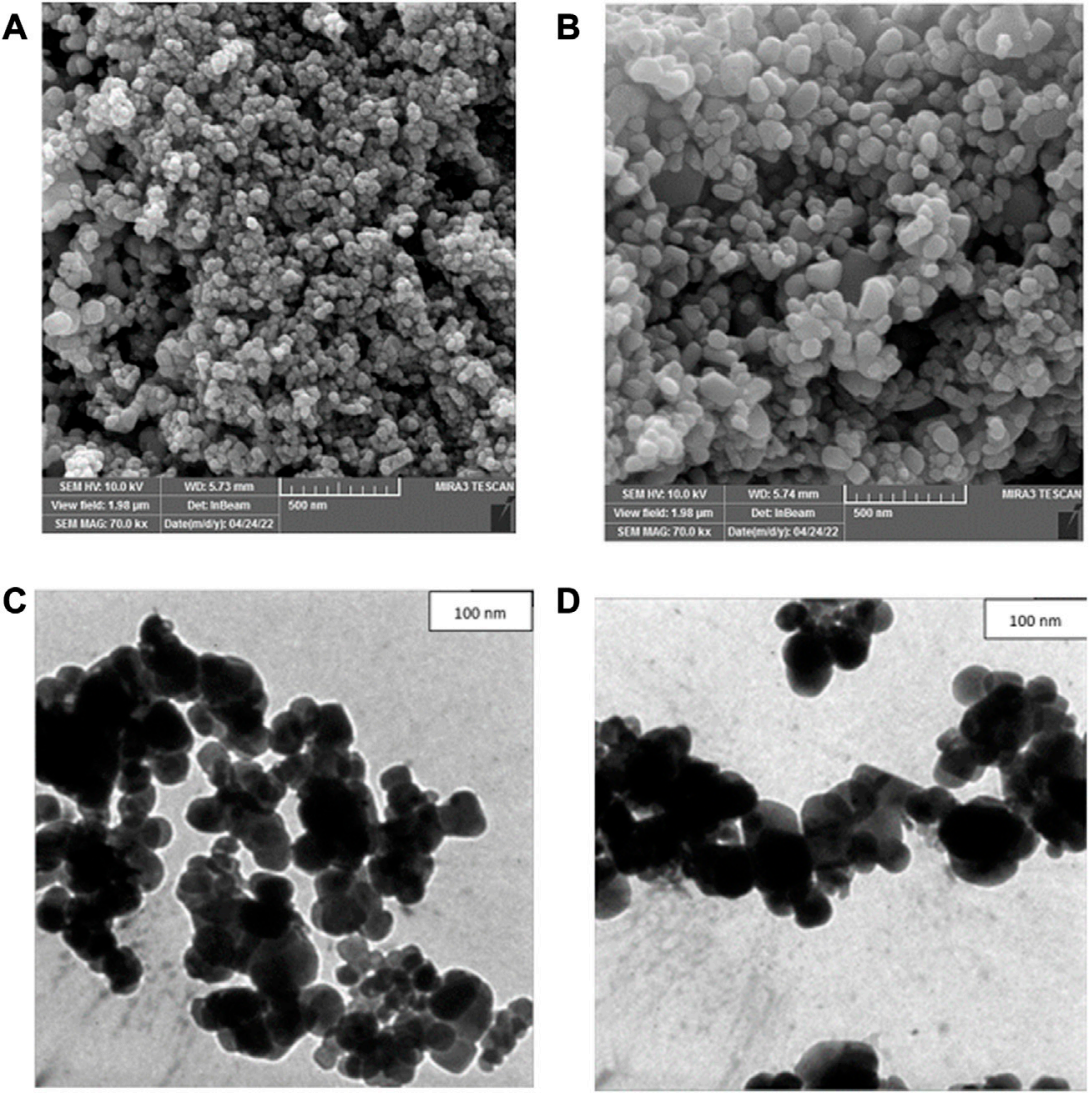
FESEM **(A, B)** and TEM **(C, D)** micrographs of crude **(A, C)** and calcined **(B, D)** CuONPs.

FESEM was used to determine morphology and size details of biogenic CuONPs. As presented in Figures 5A, B, crude and calcined CuONPs were spherical particles with the average PSD of 37.73 ± 3.27 and 48.37 ± 5.17 nm, respectively. Likewise, TEM micrographs revealed that crude and calcined CuONPs were roughly spherical particles with PSD values of 35.24 ± 4.64 and 43.68 ± 2.31 nm, respectively (Figures 5C, D).

**FIGURE 6:**
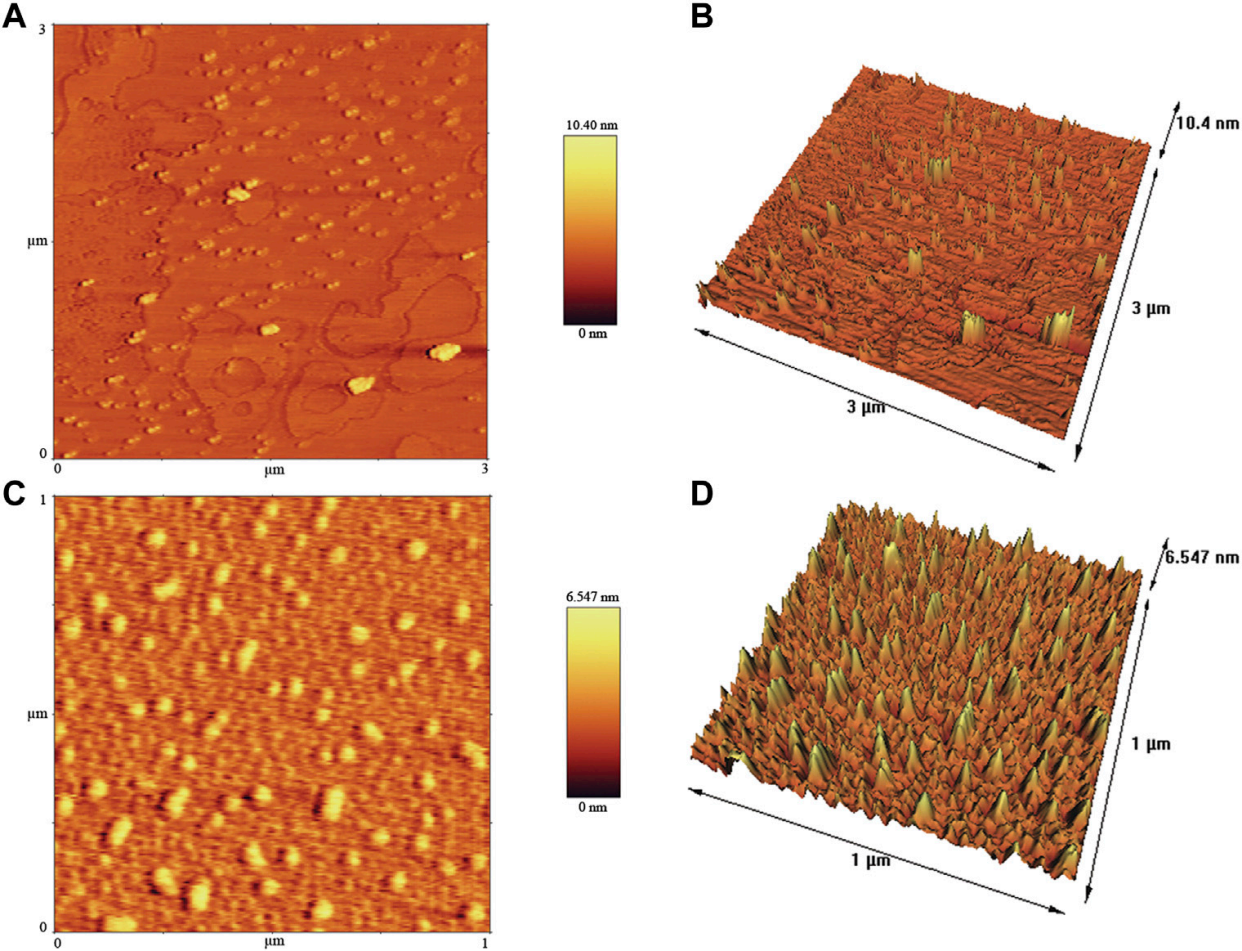
AFM images of crude **(A, B)** and calcined **(C, D)** CuONPs: 2D view **(A, C)** and 3D view **(B, D)**.

To provide further insights into topological appearance and size of CuONPs, AFM images were prepared. As presented in Figure 6, crude and calcined CuONPs were detected as individual conical grains extending upwards with the average height as 5.155 and 6.547 nm, respectively.

### Antibacterial effects of biogenic CuONPs

The antibacterial activity of CuONPs was evaluated against Gram-negative and Gram-positive bacteria using the broth microdilution method (Table 2). Calculating MIC values revealed that crude CuONPs inhibited the growth of *B. subtilis*, *S. aureus*, *P. putida* and *E. coli* at 62.5, 125, 250, and 500 μg/ml, respectively. Furthermore, the MIC values of calcined CuONPs were found to be 250 μg/ml for *B. subtilis* and *S. aureus*, and 500 and 1,000 μg/ml for *P. putida* and *E. coli*, respectively. This observation demonstrated that calcination reduced the bacterial inhibitory activity of CuONPs. As also indicated in Table 2, MBC values for crude CuONPs in the present study were 250 μg/ml for *B. subtilis, S. aureus* and *P. putida* and 500 μg/ml for *E. coli*. Likewise, the MBC values of calcined CuONPs were determined as 250 μg/ml for *B. subtilis* and *S. aureus*, and 500 μg/ml for *P. putida* and *E. coli*.

**TABLE 2:** The MIC and MBC of biogenic CuONPs against different Gram-positive and Gram-negative bacteria.

MIC (μg/ml)					MBC (μg/ml)			
	*B. subtilis*	*S. aureus*	*P. putida*	*E. coli*	*B. subtilis*	*S. aureus*	*P. putida*	*E. coli*
Crude CuONPs	62.5	125	250	500	250	250	250	500
Calcined CuONPs	250	250	500	1,000	250	250	500	500

### Antioxidant activity of biogenic CuONPs

**FIGURE 7:**
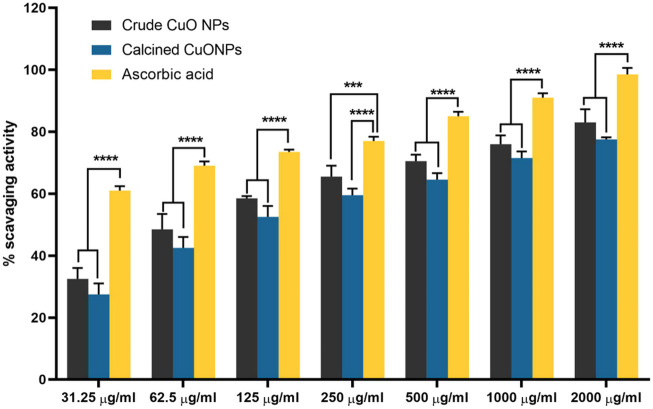
The antioxidant activity of biogenic CuONPs at different concentrations. Ascorbic acid was used as a standard. ****p < 0.0001 indicate significant difference with ascorbic acid.

Evaluating the antioxidant activity of biogenic CuONPs in the present study indicated that they scavenged DPPH radicals in a dose-dependent manner. As presented in Figure 7, upon administration of 2000 μg/ml crude CuONPs, 83% ± 2.64% antioxidant activity was detected, while calcined CuONPs exhibited lower activity (78% ± 1.73%) in the same concentration. To note, in all concentrations, significant difference (*p* < 0.0001) was detected in the scavenging activity between ascorbic acid and biogenic CuONPs.

### Anticancer properties of biogenic CuONPs

**FIGURE 8:**
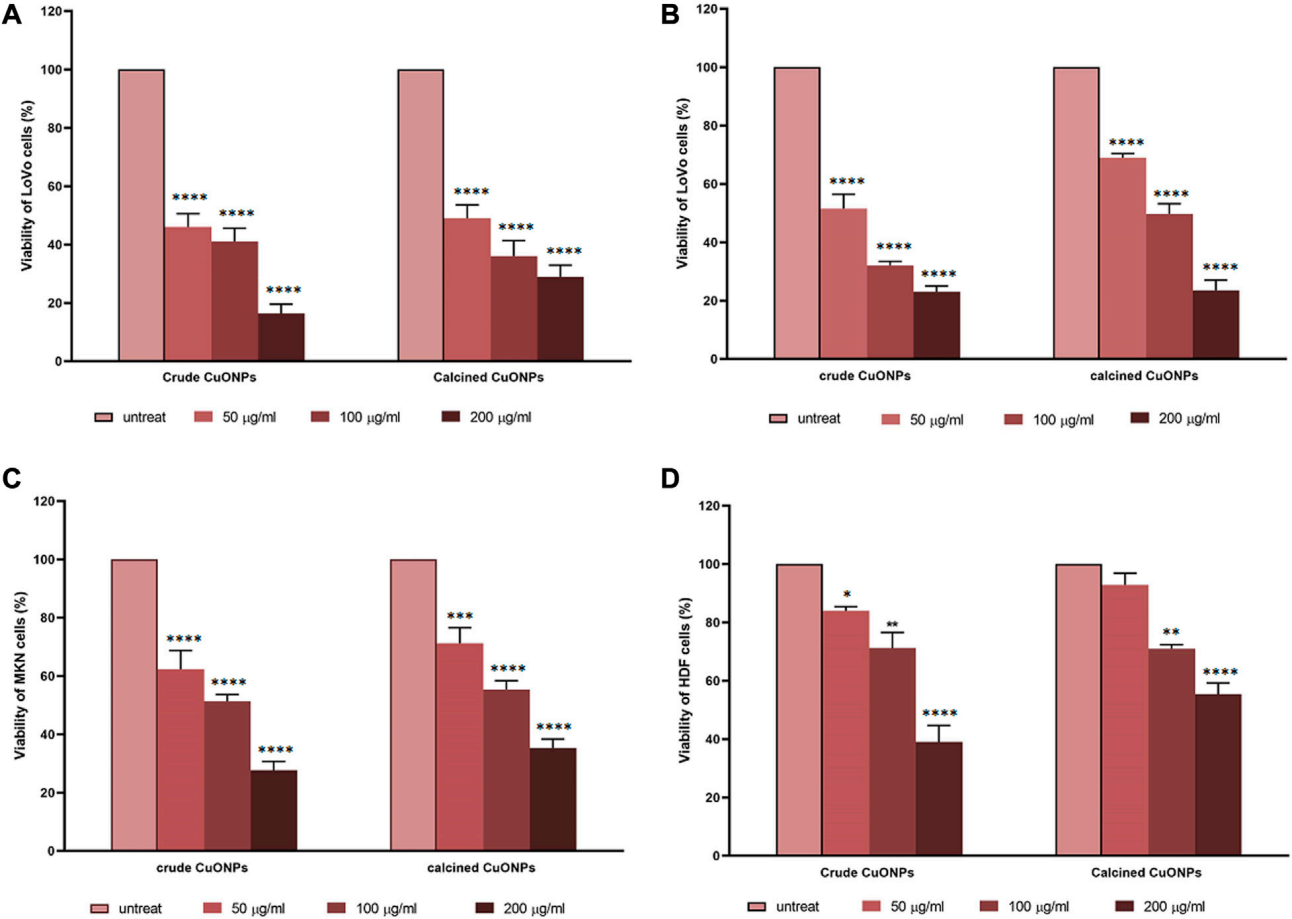
Dose-response curves representing the effects of crude and calcined CuONPs on viability of LoVo cells in normoxic **(A)** and hypoxic conditions **(B)**, MKN-45 cells **(C)**, and HDF cells **(D)**. Results are shown as mean ± SD. *p < 0.05, **p < 0.01***p < 0.001 and ****p < 0.0001 indicate significant difference with untreated cells.

**FIGURE 9:**
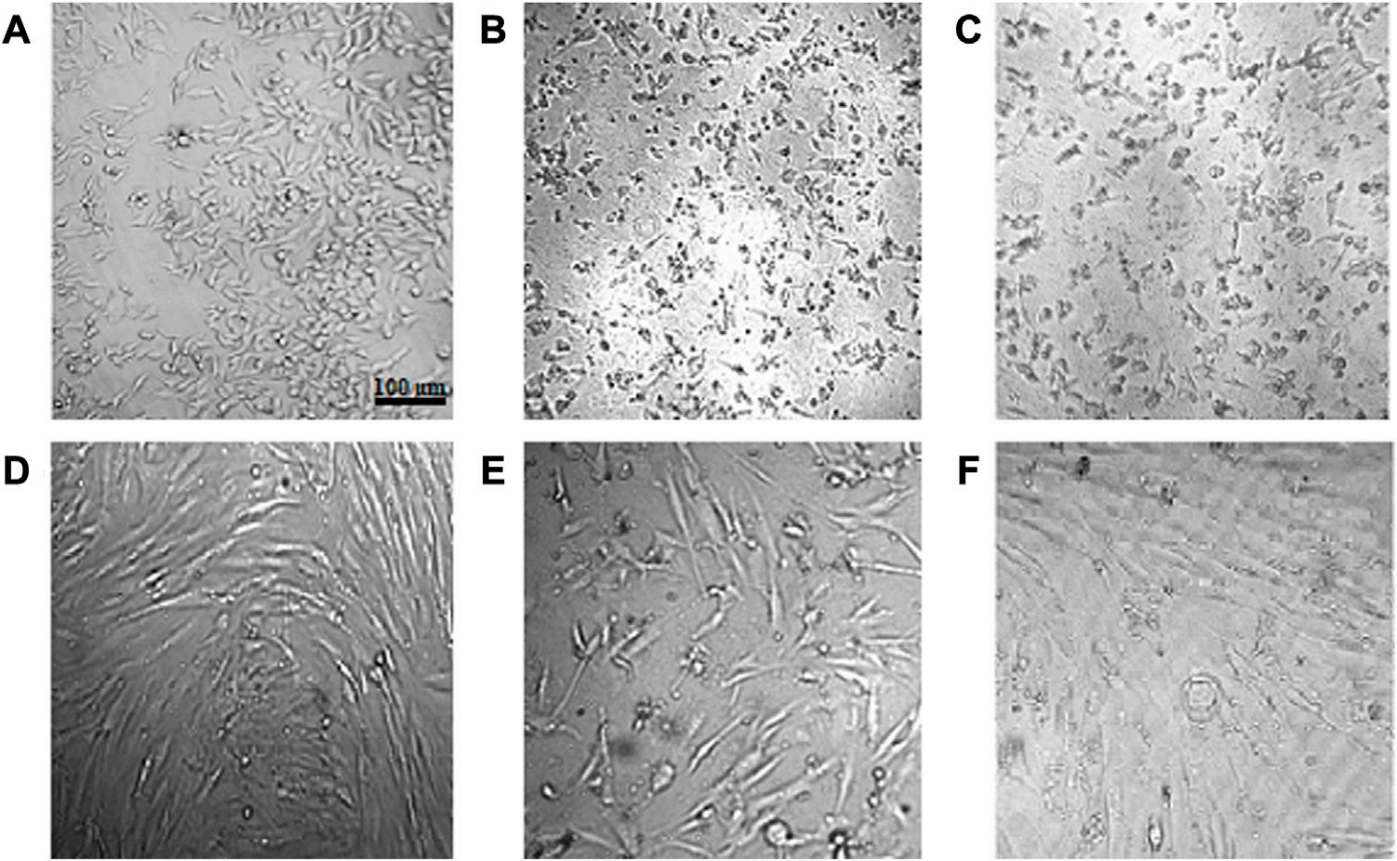
Morphological alterations of cells after administration of crude and calcined CuONPs. Phase contrast photomicrographs of LoVo **(A–C)** and HDF **(D–F)** cells; untreated **(A, D)**, treated with 200 μg/mL crude **(B, E)** and calcined **(C, F)** CuONPs.

Assessment of cell viability revealed that CuONPs induced cytotoxic effects in a dose-dependent manner. As shown in Figure 8, treatment of LoVo, MKN-45 and HDF cells with 50, 100 and 200 μg/ml crude and calcined CuONPs significantly reduced cell viability in comparison with untreated cells. To note, toxic effects of CuONPs were also cell type-dependent, as viability of LoVo cells reduced to lower amounts in comparison with MKN-45 cells, and more interestingly, our biogenic CuONPs induced lowest toxic effects on non-cancerous HDF cells. Similarly, morphological alterations, in the form of dispersed cells with cytoplasmic granulation, were apparent upon administration of crude and calcined CuONPs when compared with untreated cells (Figure 9). Calculated IC_50_ values of crude and calcined CuONPs on LoVo, MKN-45 and HDF cells are presented in Table 3. Due to high toxicity of CuONPs in LoVo cells, we also assessed their anticancer potential in hypoxic condition. Our results demonstrated that in comparison with untreated cells, biogenic CuONPs induced considerable toxicity in hypoxic condition as well (Table 3; Figure 8).

**TABLE 3:** Calculated IC_50_ (µg/ml) values of crude and calcined CuONPs on different cell lines.

	LoVo	MKN	HDF	LoVo-hypoxia
Crude CuONPs	48.36	90.23	158.2	50.84
Calcined CuONPs	44.96	117.5	222.8	92.43

**FIGURE 10:**
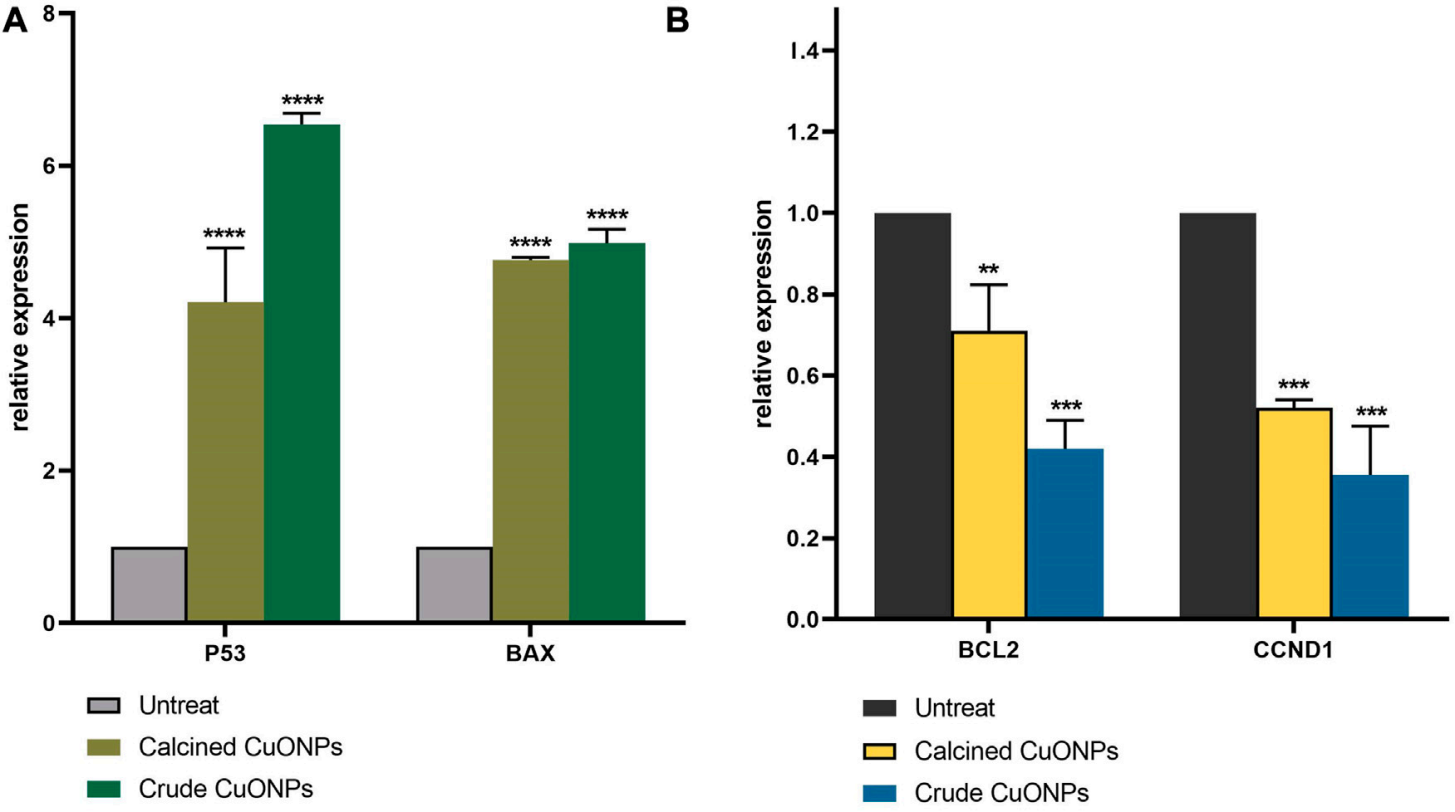
Analysis of gene expression by real-time PCR. The expression of *P53*, *BAX* **(A)**, *BCL2* and *CCND1* **(B)** were normalized and plotted as relative fold change compared with the untreated cells. Data are represented as mean ± SD. **p < 0.01, ***p < 0.001, ****p < 0.0001 indicate significant difference with untreated cells.

To unravel mechanisms underlying anticancer effects of crude and calcined CuONPs, alterations induced in the expression of apoptosis-related genes was investigated by real time PCR. As shown in Figure 10, upon 24 h treatment of LoVo cells with 100 μg/ml biogenic CuONPs, significant (*p* < 0.0001) over expression of *P53* and *BAX* was detected in comparison with untreated cells. On the other hand, crude and calcined CuONPs significantly (*p* < 0.001) downregulated the expression of *BCL2* and *CCND1* when compared with untreated cells.

## Discussion

Biosynthesis of CuONPs was carried out in the present study by an efficient non-toxic approach using *Stenotrophomonas* sp. BS95 CLS. It has been shown that cellular biomolecules such as enzymes and proteins presented in the bacterial CLS could reduce copper ions into copper atoms leading to CuONPs formation (Nakhaeepour et al., 2019; Bandeira et al., 2020). This mechanism might be involved in biosynthesis of CuONPs in our study as well. The reduction of CuSO_4_ was subjected to spectral analysis by the UV-vis spectroscopy. SPR peaks of crude and calcined CuONPs were recorded between 286 and 420 nm, respectively. In line with these findings, it has been reported that UV-vis absorbance of biogenic CuONPs fall between 285 and 570 nm (Cheirmadurai et al., 2014; Duman et al., 2016). Furthermore, changes in the SPR peak position after calcination could be explained by elimination of capping agents, increased crystallite size and/or agglomeration of metal oxide NPs, as previously described (Tang et al., 2012; Kayani et al., 2015; Gharibshahi et al., 2017).

FTIR spectrophotometry was carried out to analyze bacterial biomolecules involved in reducing copper ions to CuONPs and subsequent capping. The organic functional groups entrapping CuONPs were determined as previously reported (Ali et al., 2020; Chandrasekaran et al., 2020; Kouhkan et al., 2020). Strong peaks at 3,200–3,600 cm^-1^ demonstrated the N-H stretching vibrations of amine group or amide linkages in the protein contents of bacterial plasma membrane. The peak observed at 2,958 cm^-1^ was associated with C-H stretching vibration of the aldehyde compound. The band at 1,652 cm^-1^ was corresponded to the stretching vibration of C=O, usually found in proteins. The peaks seen at 1,080, 1,235 and 1,360 cm^-1^ were further associated with the stretching vibration of C–N of aliphatic and aromatic amines. In this study, the bands at 547 and 521 cm^-1^ for Cu–O confirmed the synthesis of CuONPs. To note, deletion of absorption peaks at 2,958 and 1,080 cm^−1^ in calcined CuONPs might be due to the removal of peaks corresponding to functional groups, including aldehyde and amine.

The presence of sharp structural peaks in XRD patterns and crystallite size <100 nm revealed the nanocrystalline nature of crude and calcined CuONPs, which are in consistence with previous studies (Ahamed et al., 2014; Shankar and Rhim, 2014; Duman et al., 2016). Metal oxide NPs with zeta potential values higher than +30 mV or lower than −30 mV typically have high degree of stability, which is of utmost importance to avoid their agglomeration in colloidal solution (Joseph and Singhvi, 2019). In the present study, zeta potential measurement indicated higher stability of crude and calcined CuONPs compared to previous reports (Tiwari et al., 2016; Chandrasekaran et al., 2020).

Defining the morphology and PSD of biogenic CuONPs by FESEM, TEM and AFM revealed their spherical shape, while crude CuONPs presented smaller size in comparison with calcined CuONPs. These observations confirmed results obtained from XRD and DLS analysis, and are in consistence with previous reports on biogenic CuONPs (Nasrollahzadeh et al., 2015; Gu et al., 2018; Kouhkan et al., 2020; Sarkar et al., 2020).

Evaluating antibacterial activity of CuONPs implied on their inhibitory effects on both Gram-negative and Gram-positive pathogens, although antibacterial potential of crude CuONPs was higher than calcined CuONPs. In consistence with current findings, previous reports indicated that metal oxide NPs such as CuONPs induced remarkable antimicrobial activity due to their small size and extremely large surface area that provide better contact with microorganisms (Azam et al., 2012; Laha et al., 2014). Various studies have also demonstrated bactericide effects of CuONPs against same pathogenic bacteria, for instance, MBC values of crude CuONPs on *E. coli* and *S. aureus* have been reported as 250 and 2,500 μg/ml, respectively (Ren et al., 2009). In addition, MBC of mechanochemically synthesized CuONPs against *E. coli* and *S. aureus* were as 750 and 5,000 μg/ml, respectively (Moniri et al., 2019). In another research, the MBC of phytofabricated CuONPs was reported as 10,000 μg/ml for both *E. coli* and *S. aureus* (Alavi et al., 2021). Based on our findings, biogenic CuONPs induced higher growth inhibitory and toxic effects on Gram-positive bacteria compared with Gram-negative ones. In this regard, it has been shown that lipopolysaccharide layer on the outer membrane of Gram-negative bacteria acts as an effective protection against NPs (Franco et al., 2022) that could explain, to some extent, observed effects in the present study. CuONPs induce antibacterial effects through binding to the bacterial cell membrane, production of reactive oxygen species and release of Cu^2+^ ions that demolish DNA and cellular proteins, affect the membrane permeability, and finally induce cell death (Obeizi et al., 2020). Thus, considerable activity of biogenic CuONPs against *B. subtilis, S. aureus, P. putida, and E. coli* in our study might be mediated through the same mechanisms.

Current results also revealed high antioxidant activity of crude and calcined CuONPs, which has been attributed to the binding of transition metal ion catalysts to free radicals (Omran and Baek, 2021). Based on a recent report, the free radical scavenging activity of CuONPs may be enhanced by various bio-reductive groups (capping agents) of the bacterial proteins (Ssekatawa et al., 2022). As explained above, FTIR analysis confirmed the capping of crude CuONPs with aldehyde and amine groups, unlike calcined CuONPs. Therefore, higher antioxidant activity of crude CuONPs in our study was presumably due to bacterial-derived functional groups.

Malignancies of the gastrointestinal tract, including colorectal and gastric carcinomas, account for 36.2% of cancer mortality (Hong et al., 2022). Although use of chemical drugs is a systemic treatment for cancer patients, low specificity of common chemotherapeutics causes many side effects that are mostly intolerable to patients and lead to reduced survival rates (Pearce et al., 2017; Fu et al., 2018; Zhang et al., 2018). To introduce novel and more effective therapeutics, we investigated anticancer potential of CuONPs, and obtained findings revealed that cytotoxicity of our biogenic CuONPs was dose- and cell type-dependent. Intriguingly, crude and calcined CuONPs induced more toxic effects on human colon and gastric adenocarcinoma cells than normal fibroblasts. Current results are in consistence with previous reports. For instance, it has been demonstrated that CuONPs synthesized by marine endophytic actinomycete and *Vibrio* sp. VLC. induced toxic effects on human lung and esophageal carcinoma cells in a dose-dependent manner, with IC_50_ values of 500 and 37.52 μg/ml, respectively (Nakhaeepour et al., 2019; Zhao et al., 2022). In addition, low toxicity of CuONPs on normal HDF cells in the present study is in line with another research, which reported minimal toxic effects of CuONPs on human dermal fibroblasts (Sulaiman et al., 2018).

Hypoxia, a biological phenomenon in which oxygen level is below the tissue demand, is a feature of solid tumors, and an indicator of poor prognosis in many cancers including colon and gastric adenocarcinomas (Qi et al., 2020; Pei et al., 2021). Hypoxia causes a range of genetic, transcriptional, and metabolic adaptations in advanced tumors that ultimately promote survival and metastasis of cancer cells (Li et al., 2020). Thus, it has been recommended to assess anticancer effects of potent agents in hypoxic condition for accurate evaluation of their therapeutic potential (Nobre et al., 2018). Current findings that revealed considerable cytotoxicity of biogenic CuONPs in hypoxic condition suggest that these NPs have the potential to induce anticancer effects *in vivo*, although more research on animal models is required.

Carrying gene expression analysis indicated significant induction in *P53* expression after administration of biogenic CuONPs. *P53* is a tumor suppressor gene with critical roles in the cell cycle regulation and apoptosis (Vousden and Lu, 2002; Lacroix et al., 2006). Similar to our results, it has been reported that CuONPs induced apoptosis in human lung carcinoma cells through upregulation of *P53* (Kalaiarasi et al., 2018). In the present study, CuONPs effectively downregulated the expression of *BCL2,* while induced the expression of *BAX*. Current findings are in agreement with previous studies, which demonstrated that CuONPs inhibited cell growth and induced apoptosis in acute myeloid leukemia, breast, gastric, colon and lung cancer cells *via* significant induction of *BAX* and downregulation of *BCL2* (Shafagh et al., 2015; Gopinath et al., 2016; Khan et al., 2017; Kalaiarasi et al., 2018). Present results also revealed that CuONPs significantly decreased the expression of *CCND1*, a core cell cycle regulator that promotes cell proliferation and plays a major role in oncogenesis (Qie and Diehl, 2016). Similar to our findings, it has been shown that inhibited proliferation and induced apoptosis of oral carcinoma cells upon administration of metal oxide NPs were mediated by downregulation of *CCND1* (Li et al., 2020).

## Conclusion

Emergence of antibiotic-resistant strains is an intractable challenge to public health worldwide. In addition, acquired chemoresistance of cancer cells has vastly limited the clinical outcome of current pharmaceutical drugs. Although inorganic NPs could act as potent antibiotics and anticancer agents, disadvantages of conventional methods for NP synthesis have enforced to develop alternative approaches. In the present attempt, we successfully used *Stenotrophomonas* sp. BS95 to synthesize functional CuONPs. UV vis spectroscopy, XRD and DLS analyses and electron microscopy revealed small size, crystallin nature and spherical shape of CuONPs. In addition, good dispersion and high stability of biogenic CuONPs were confirmed by zeta potential analysis, and functional groups were determined by FTIR spectroscopy. Evaluating biological effects of CuONPs exhibited their antibacterial activity against *B. subtilis*, *S. aureus*, *P. putida*, and *E. coli*. Furthermore, biogenic CuONPs possessed remarkable antioxidant potential and induced considerable anticancer effects on human colon and gastric adenocarcinoma cells *via* modulation of apoptosis-related genes. Interestingly, biogenic CuONPs induced low toxicity on normal cells, and had the potential to exert cytotoxic effects in hypoxic condition. According to the current findings, our biogenic CuONPs could be considered as effective agents with potential medical applications. Nevertheless, complementary studies on other pathogenic bacteria, more cell lines and animal models are required to better evaluate the efficacy and safety of biogenic CuONPs. In addition, conjugation of biogenic CuONPs with antibiotics and anticancer drugs might improve the clinical outcome of current therapeutic modalities.

## Data Availability

The original contributions presented in the study are included in the article/supplementary material, further inquiries can be directed to the corresponding author.
